# Glucose fluctuations aggravate myocardial fibrosis via activating the CaMKII/Stat3 signaling in type 2 diabtetes

**DOI:** 10.1186/s13098-023-01197-5

**Published:** 2023-10-28

**Authors:** Lei Zhang, Huan-Huan Liu, Fan Yang, Zhi-Yuan Zhang, Zhen-Ye Zhang, Xiao-Xi Zhao, Ling-Ling Qian, Shi-Peng Dang, Ru-Xing Wang

**Affiliations:** 1 Department of Cardiology, Wuxi People’s Hospital of Nanjing Medical University, Wuxi Medical Center, Nanjing Medical University, Wuxi, 214023 China; 2 Wuxi School of Medicine, Jiangnan University, Wuxi, 214122 China

**Keywords:** Glucose fluctuations, Myocardial fibrosis, CaMKII, Stat3

## Abstract

**Background:**

Glucose fluctuations (GF) are a risk factor for cardiovascular complications associated with type 2 diabetes. However, there is a lack of adequate research on the effect of GF on myocardial fibrosis and the underlying mechanisms in type 2 diabetes. This study aimed to investigate the impact of glucose fluctuations on myocardial fibrosis and explore the potential mechanisms in type 2 diabetes.

**Methods:**

Sprague Dawley (SD) rats were randomly divided into three groups: the control (Con) group, the type 2 diabetic (DM) group and the glucose fluctuations (GF) group. The type 2 diabetic rat model was established using a high-fat diet combined with low-dose streptozotocin injection and the GF model was induced by using staggered glucose and insulin injections daily. After eight weeks, echocardiography was used to assess the cardiac function of the three groups. Hematoxylin-eosin and Masson staining were utilized to evaluate the degree of pathological damage and fibrosis. Meanwhile, a neonatal rat cardiac fibroblast model with GF was established. Western and immunofluorescence were used to find the specific mechanism of myocardial fibrosis caused by GF.

**Results:**

Compared with rats in the Con and the DM group, cardiac function in the GF group showed significant impairments. Additionally, the results showed that GF aggravated myocardial fibrosis in vitro and in vivo. Moreover, Ca^2+^/calmodulin‑dependent protein kinase II (CaMKII) was activated by phosphorylation, prompting an increase in phosphorylation of signal transducer and activator of transcription 3 (Stat3) and induced nuclear translocation. Pretreatment with KN-93 (a CaMKII inhibitor) blocked GF-induced Stat3 activation and significantly suppressed myocardial fibrosis.

**Conclusions:**

Glucose fluctuations exacerbate myocardial fibrosis by triggering the CaMKII/Stat3 pathway in type 2 diabetes.

**Supplementary Information:**

The online version contains supplementary material available at 10.1186/s13098-023-01197-5.

## Introduction

Diabetes is a common chronic disease manifesting as chronic persistent hyperglycemia and glucose fluctuations (GF). Glucose fluctuations, also known as glucose variability, are developing as an emerging indicator of glycemic control [1, 2]. Previous studies have shown that a higher GF is independently associated with heart failure [3], arrhythmias [4], and cardiovascular events [5]. In type 1 diabetes, GF can lead to myocardial fibrosis [6]; however, there is currently limited understanding of whether GF can lead to myocardial fibrosis in type 2 diabetes and the mechanisms involved have not been clarified.

Ca^2+^/calmodulin‑dependent protein kinase II (CaMKII), a multifunctional serine/threonine-protein kinase, has been identified as a critical factor in various cardiac diseases [7–9]. Many studies have shown that diabetes can increase CaMKII activity in the myocardium [10], resulting in ROS induction [11], necroptosis [12] and ion channel anomalies and so on. Furthermore, Das et al. [13] demonstrated that CaMKII inhibition can prevent chemotherapy-induced cardiac fibrosis. However, the potential effects of CaMKII in myocardial fibrosis resulting from GF are still unknown.

Signal transducer and activator of transcription 3 (Stat3), one of the STAT members, has increasingly gained focused attention due to its significant roles in metabolic diseases [14], cardiac hypertrophy [15, 16], heart failure [17] and arteriosclerosis [18]. Previous studies have verified that inhibition of p-Stat3 attenuated cardiomyopathy caused by type 1 diabetes [19]. Additionally, Unudurthi et al. have found that CaMKII and Stat3 can interact with each other [20]. However, the role of Stat3 activation in myocardial fibrosis induced by GF and upstream events leading to Stat3 activation still needs to be elucidated. Hence, the present study was carried out to unravel the effect of glucose fluctuations on myocardial fibrosis in type 2 diabetes and explore the underlying mechanisms.

## Methods

### Experimental animals

Male 6–8 weeks Sprague–Dawley (SD) rats were purchased from Changzhou Cavins Biotechnology Company. The rats were housed in a standard environment with 23 ± 1^◦^C, 55–65% humidity and under a 12-h light/12-h dark cycle. All rats were fed with the normal diets and water freely. After one week of adaptive feeding, the rats were randomly divided into two groups, the control (Con) group (n = 15) and the type 2 diabetic (T2DM) group. The Con group rats were fed with normal chow. The T2DM model was established by a low dose (35 mg/kg) of streptozotocin (STZ, Sigma-Aldrich, S0130) intraperitoneally after four weeks on a high-fat and high-sugar diet (D12492, Suzhou SPF Biotechnology Co., Ltd, China), according to the method used in previous studies [21]. Blood was extracted from the tail vein after three days and blood glucose > 16.7 mmol/L was considered successful in modeling. Then, T2DM rats were divided into the diabetic (DM) group (n = 15) and the GF group (n = 15). The rats in the DM group continued to be fed with the high-fat diet. The GF model was established based on the previous literature [22]. In brief, the rats in the GF group were injected with insulin subcutaneously at 8:00, 12:00 and 16:00 daily, and 3 g/kg glucose was injected intraperitoneally at 10:00, 14:00 and 18:00 daily for eight weeks. Blood glucose was measured in the tail vein 30 min after each insulin or glucose injection. All animal experiments complied with the Guide for the Care and Use of Laboratory Animals (the revised Animals (Scientific Procedures) Act 1986) and were approved by the Ethics Committee of the Affiliated Wuxi People’s Hospital of Nanjing Medical University.

### Echocardiography evaluation

After eight weeks, heart function was evaluated using echocardiography (Philip, ie33). After being anesthetized with isoflurane (2%), the hair on the chest of the rats in the three groups (n = 6 per group) was shaved and acoustic coupling gel was then applied. M mode of echocardiography was performed to record the following parameters: left ventricular percent ejection fraction (EF), left ventricular fractional shortening (FS), left ventricular internal diameter at end-diastole (LVIDd), and left ventricular internal diameter at end-systole (LVIDs).

### Histopathological analysis

After the rats were sacrificed, the left ventricular tissue samples were fixed for 24 h in 4% paraformaldehyde, then paraffin-embedded and sliced into 4-µm sections for hematoxylin-eosin (HE, Beyotime, C0105) and Masson staining (Nanjing Jiancheng Bioengineering Institute, D026). Afterward, the sections were observed under a light microscope (DP73, OLYMPUS).

### Primary culture of neonatal rat cardiac fibroblasts

Neonatal rat cardiac fibroblasts (NRCFs) were extracted using the previous method [6]. Neonatal rats born 1–3 days old were selected, and the hearts were quickly removed and digested several times at 37 °C using 0.125% trypsin (Gibco, 25200072) and 0.1% collagenase (Worthington, LS004176). The cell suspension was then seeded in DMEM mediums containing 10% fetal bovine serum (FBS, Gibco, 12664025). After 1 h, remove the unadhered cells and add new mediums. The cells were incubated in a 37 °C incubator with 5% CO_2_.

### Glucose fluctuations cell model and treatments

NRCFs were divided into three groups as previously reported: the normal glucose (Ctrl) group, the high glucose (HG) group and the glucose fluctuations (GF) group. Cells in the Ctrl group were cultured using DMEM containing 5.5 mmol/L glucose, cells in the HG group were cultured in DMEM containing 33 mmol/L glucose, and cells in the GF group were cultured in DMEM containing 5.5 mmol/L and 33 mmol/L glucose alternately every 12 h for 72 h. In addition, to confirm the effect of CaMKII on myocardial fibrosis, the NRCFs were also treated with KN-93 (a CaMKII inhibitor, 0.5 µmol/L, Medchemexpress, HY-15465) [23].

### Western blot analysis

Rat ventricular myocardial tissue or fibroblasts were lysed in lysis buffer containing a cocktail of proteinase/phosphatase inhibitors and then centrifuged at 12000 rpm for 15 min at 4 °C. The proteins were then transferred to PVDF membranes using SDS-PAGE. The PVDF membrane was then incubated with primary antibodies of p-CaMKII (Abcam, ab32678), CaMKII (Santa Cruz, sc100362), p-Stat3 (Cell Signaling Technology, 9145S), Stat3(Cell Signaling Technology, 12640S), Collagen I (Proteintech, 14695-1-AP), Collagen III (Proteintech, 22734-1-AP), TGF-β1 (Abcam, ab179695), β-actin (Abcam, ab6276), β-tubulin(Abcam, ab21058) at 4 °C overnight followed by corresponding secondary antibody incubation. Blot bands were quantified using the Image J software.

### Immunofluorescence analysis

Neonatal rat cardiac fibroblasts were washed with phosphate buffered saline (PBS) and fixed using 4% paraformaldehyde for 15 min. Then, the cells were permeabilized with 0.5% Triton X-100 (Sigma-Aldrich, T9284) and blocked with goat serum (Solarbio, SL038) in PBS for 30 min at 37 °C. After that, NRCFs were incubated at 4 °C overnight with the primary rabbit antibody against p-Stat3, and a fluorescence secondary antibody was added for incubation of 1 h at 37 °C. Afterward, the cells were washed with PBS and counterstained the nucleus with DAPI (Beyotime, C1005) for 10 min and analyzed under a fluorescence microscope.

### Statistical analysis

Data were shown as mean ± standard error of mean (SEM). Shapiro-Wilk normality test was used to test the data distribution. Non-normally distributed data were analyzed by nonparametric tests. Normally distributed data were analyzed by One-way ANOVA with post hoc LSD. *P* < 0.05 was considered statistically significant. All statistics were determined using SPSS 25.0 software.

## Results

### Glucose fluctuations exacerbated the impairment of cardiac dysfunction

**Fig. 1:**
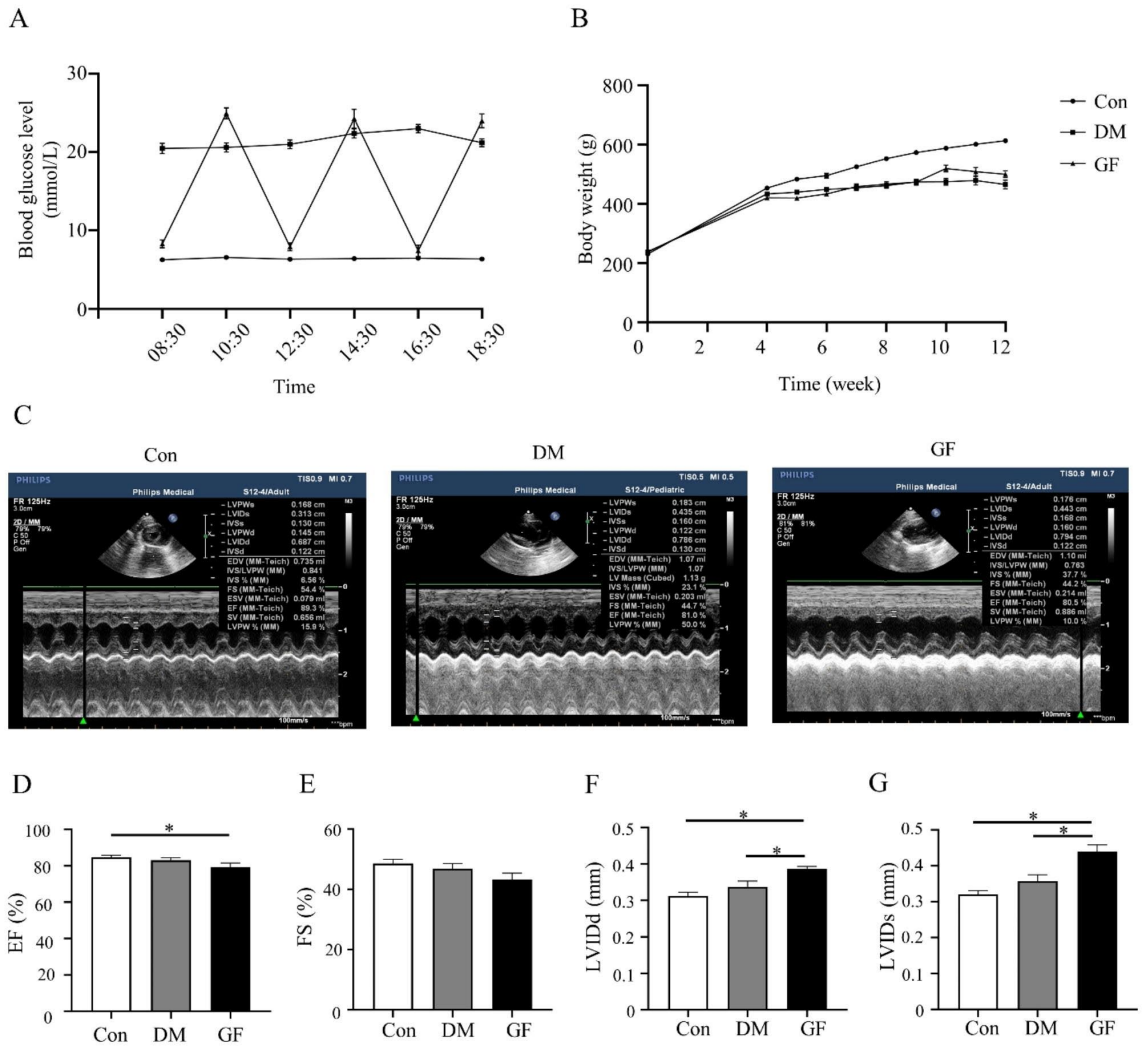
Glucose fluctuations accelerated cardiac dysfunction. (**A**) Daily blood glucose levels in three groups of rats (n = 15). (**B**) Body weight levels in the three groups (n = 15). (**C**) Representative echocardiographic images of rats. (**D**-**G**) Measurements of ejection fraction (EF), fractional shortening (FS), left ventricular internal diameter at end-diastole (LVIDd) and left ventricular internal diameter at end-systole (LVIDs) (n = 6)

The blood glucose of rats in the three groups 30 min after glucose, insulin injection or normal saline solution was shown in Fig. 1A. Rats in the GF and DM groups had significantly lower body weight than those in the Con group (Fig. 1B). In addition, this study also showed that GF exacerbated cardiac dysfunction by suppressing the EF and increasing the LVIDd and LVIDs (Figures C-G).

### Glucose fluctuations aggravated myocardial fibrosis

**Fig. 2:**
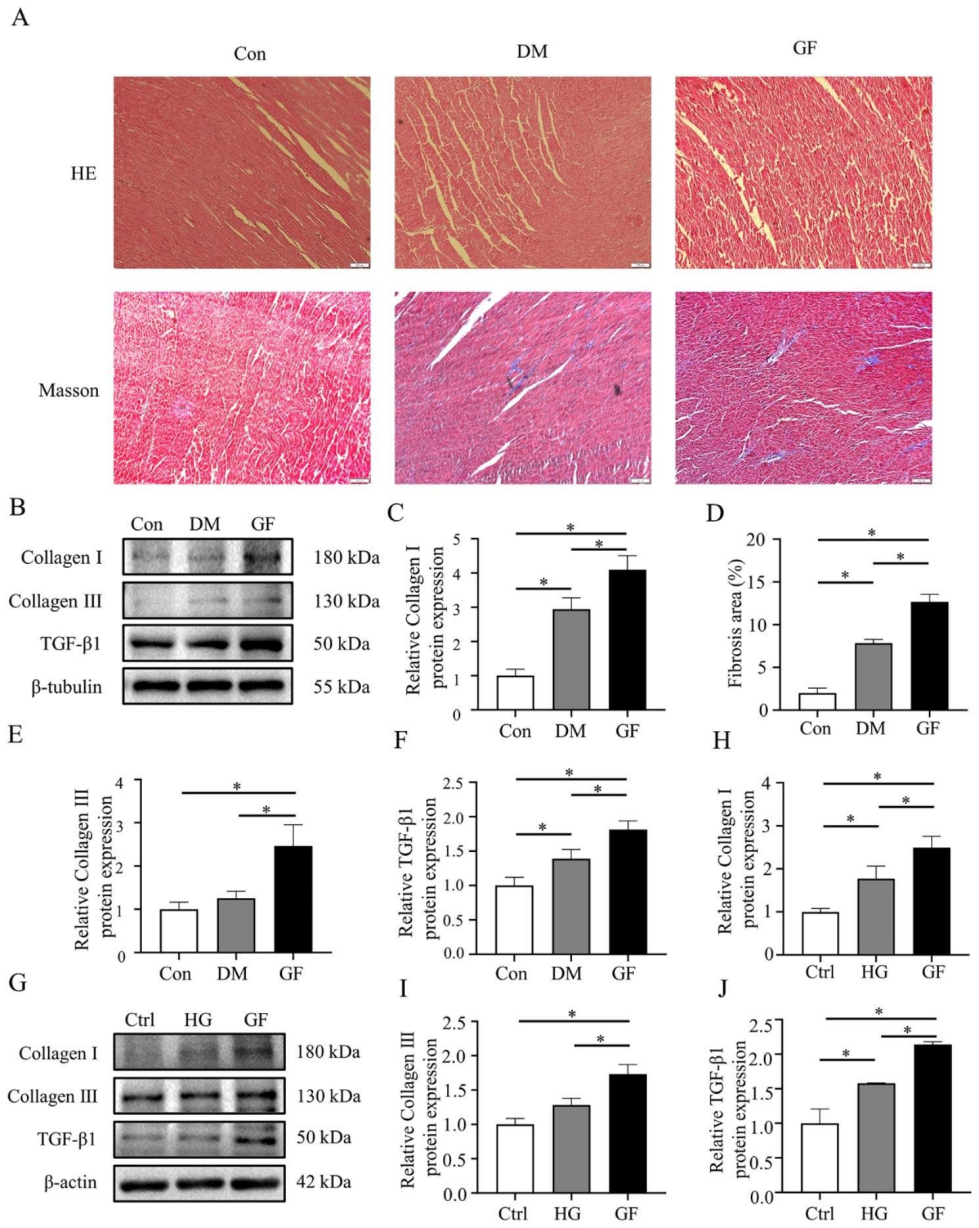
Glucose fluctuations promoted myocardial fibrosis. (**A**, **D**) HE and Masson staining of ventricular muscle tissue from three groups of rats (n = 3). (**B**, **C**, **E**, **F**) The protein expressions of Collagen I (n = 5), Collagen III (n = 5) and TGF-β1 (n = 6) in rat hearts of the three groups. (**G**-**J**) The protein expressions of Collagen I (n = 6), Collagen III (n = 5) and TGF-β1 (n = 4) in NRCFs of the three groups

The HE staining of the three groups showed that the arrangement of myocardial fibers was disordered and there were myocardial fiber breaks in the GF compared to the Con and DM groups. Moreover, the Masson staining showed a significant increase in fibrosis in the ventricular tissue of the GF group rats (Fig. 2A and D). Additionally, GF can significantly increase the protein expression of Collagen I, Collagen III and TGF-β1 in vitro and in vivo (Fig. 2B-C and E-J). Those results indicated that GF can lead to myocardial fibrosis.

### Glucose fluctuations promoted the activation of CaMKII and Stat3

**Fig. 3:**
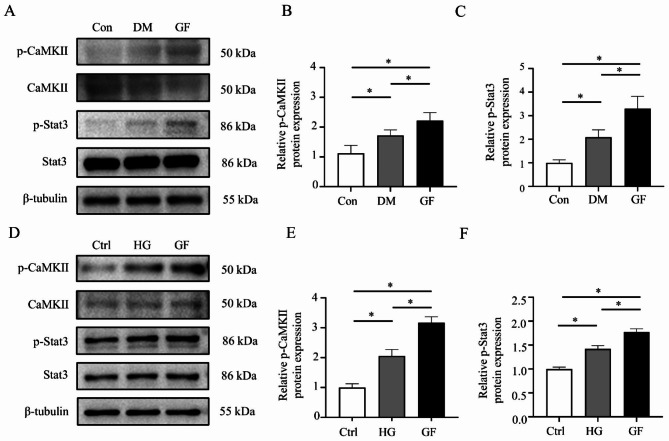
Glucose fluctuations upregulated the expression of phosphorylated CaMKII and Stat3. (**A**-**C**) The protein expressions of phosphorylated CaMKII (n = 5) and Stat3 (n = 6) in rat hearts of the three groups. (**D**-**F**) The protein expressions of phosphorylated CaMKII (n = 3) and Stat3 (n = 6) in NRCFs of the three groups

**Fig. 4:**
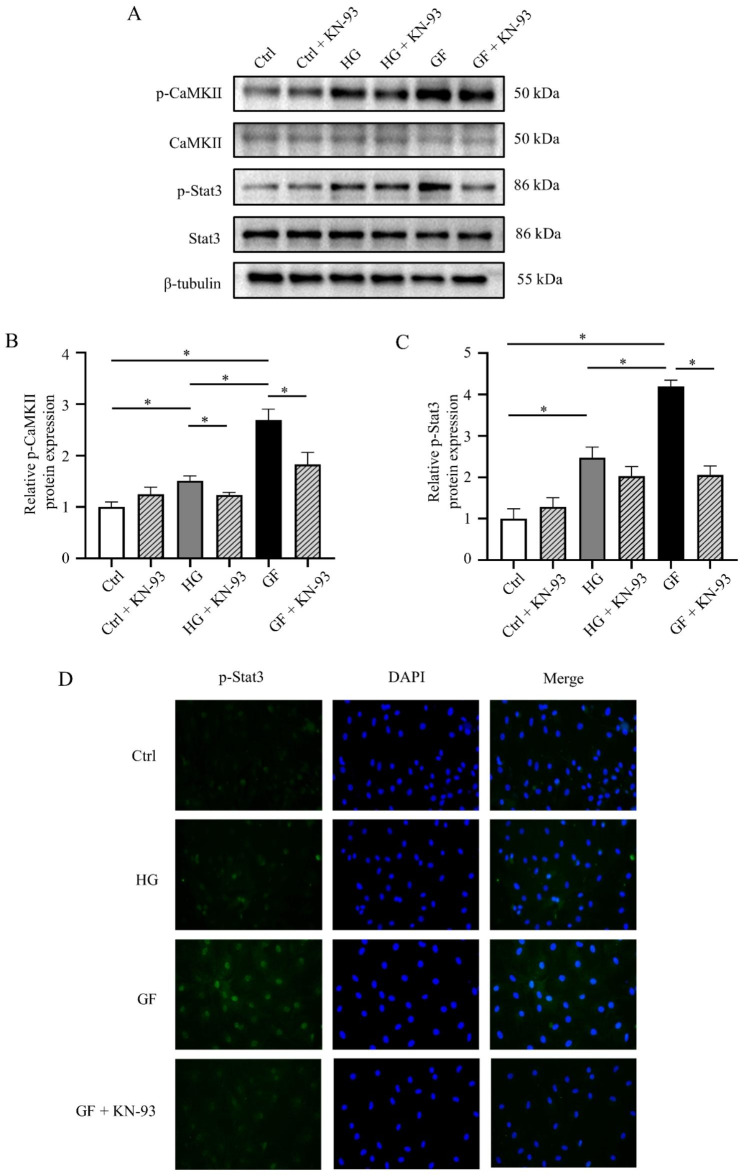
Inhibition of CaMKII attenuated p-Stat3 elevation due to glucose fluctuations. (**A**) Representative bands of Western blotting of p-Stat3 in NRCFs after using KN-93. (**B**-**C**) Relative levels of p-Stat3 in three groups after using KN-93 (n = 4). (**D**) Immunofluorescence staining of nuclear translocation of p-Stat3 in three groups

To determine whether CaMKII and Stat3 were activated in GF-induced myocardial fibrosis, the relative expressions of total and phosphorylated*/*activated forms of CaMKII and Stat3 were tested. The results showed upregulation of CaMKII phosphorylation and Stat3 phosphorylation protein expression in the GF rats (Fig. 3A-C) and the cells with fluctuated glucose concentrations (Fig. 3D-F). Immunofluorescent staining showed that GF increased p-Stat3 nuclear accumulation compared to the Con and DM groups (Fig. 4D).

### Inhibition of CaMKII reduced myocardial fibrosis

**Fig. 5:**
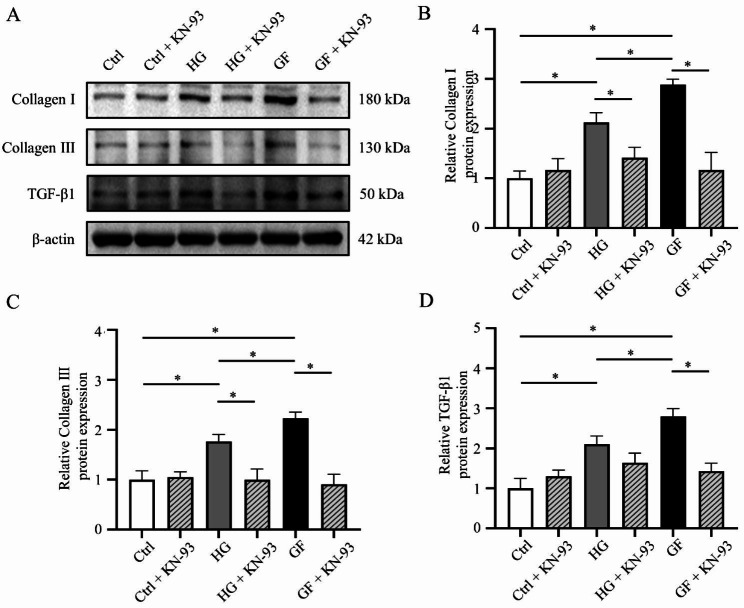
Role of CaMKII in glucose fluctuation-induced myocardial fibrosis. (**A**) Representative bands of Western blotting of Collagen I, Collagen III and TGF-β1 in NRCFs after using KN-93. (**B**-**D**) Relative levels of Collagen I, Collagen III and TGF-β1 in three groups after using KN-93 (n = 4)

To investigate the role of CaMKII in GF-induced myocardial fibrosis, we used a CaMKII-specific inhibitor, KN-93. As shown in Fig. 5, KN-93 can significantly reduce the expression of Collagen I and Collagen III in HG and GF groups compared with the Con group (Fig. 5A-C). Moreover, the inhibition of CaMKII reversed the upregulation of TGF-β1 in GF groups (Fig. 5A and D). The above results suggested that inhibition of CaMKII can attenuate myocardial fibrosis caused by GF.

### Activation of CaMKII promoted GF-induced myocardial fibrosis via activating Stat3

To determine whether Stat3 was the downstream target of CaMKII, we examined the expression of phosphorylated Stat3 in the three groups. The results revealed that inhibition of CaMKII decreased the phosphorylation of Stat3 (Fig. 4A-C). In addition, an immunofluorescence assay was used to evaluate the nuclear translocation of p-Stat3. After the application of KN-93, highly expressed phosphorylated Stat3 in the nucleus of NRCFs in the GF group can be reversed (Fig. 4D).

## Discussion

**Fig. 6:**
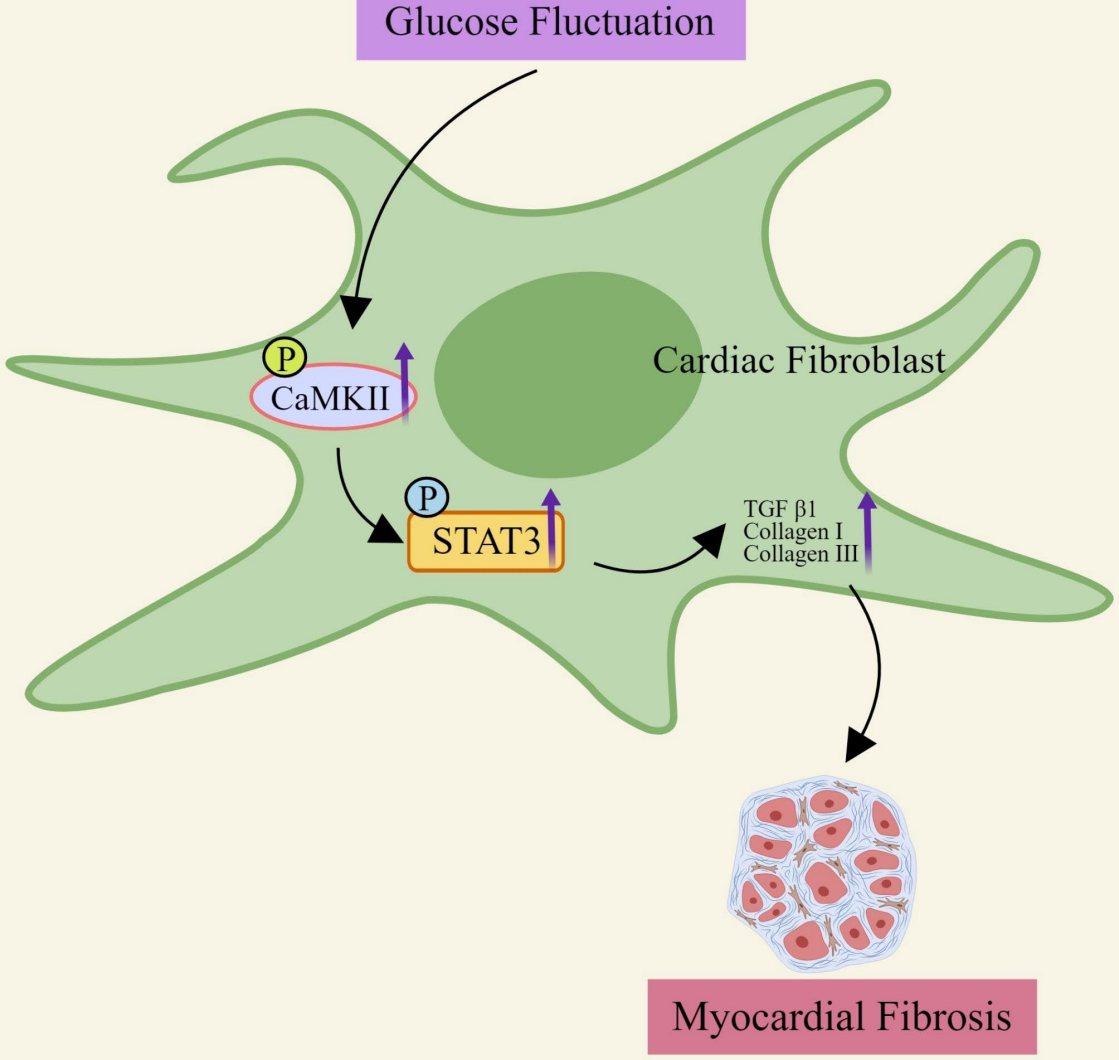
Role of CaMKII/Stat3 pathway in myocardial fibrosis induced by glucose fluctuation. Glucose fluctuation contributes to the upregulation of CaMKII phosphorylation, leading to Stat3 phosphorylation, and increased expression of fibrosis-related proteins.CaMKII, Ca^2+^/calmodulin‑dependent protein kinase II; Stat3, Signal transducer and activator of transcription 3

Glycemic management in diabetic patients is now focused not only on effective glucose reduction, but also on how to avoid glucose fluctuations [1]. Our previous studies have shown that glucose fluctuations contribute to the development of many cardiovascular diseases [5, 24]. More importantly, GF in type 1 diabetes can lead to increased myocardial fibrosis [6], but the pathophysiological mechanisms contributing to GF-induced myocardial fibrosis remain elusive, especially in type 2 diabetes. Here, we demonstrated that GF in type 2 diabetes can increase myocardial fibrosis. CaMKII activation played an essential role in this process by activating Stat3, which led to increased myocardial fibrosis (Fig. 6).

As widely reported, CaMKII is a multifunctional serine, threonine protein kinase with four isoforms, α, β, γ and δ. The δ isoform is predominantly expressed in the myocardium and is involved in the development of various cardiovascular diseases, especially in electrical remodeling [9, 10, 25, 26]. However, studies on the role of CaMKII in the development of diabetic myocardial fibrosis are limited, especially in diabetic glucose fluctuations. This study showed that GF in type 2 diabetes activated CaMKII, leading to the development of myocardial fibrosis. In contrast, the use of the CaMKII inhibitor KN-93 attenuated myocardial fibrosis. These data demonstrated the vital role of CaMKII in myocardial fibrosis due to GF in type 2 diabetes. Similarly, a previous report has shown that inhibition of CaMKII can attenuate myocardial fibrosis caused by chemotherapy [13]. In addition, upregulation of BACH1 mediated activation of CaMKII was proven to accelerate cardiac hypertrophy and fibrosis [27]. In the current study, it was observed that hesperidin, a specific small-molecule inhibitor of CaMKII-δ, directly bound to CaMKII-δ and specifically blocked its activation in an ATP-competitive manner, may provide a strategy for the joint therapy of cardiovascular disease [28].

Stat3 can be activated through multiple mechanisms and translocated to the nucleus, where it acts as a transcription factor and cofactor [20]. Recent studies have revealed that high glucose can activate Stat3 in fibroblasts, and promote their proliferation and migration [29]. Similarly, high-concentration glucose can induce EGFR-mediated Stat3 phosphorylation, and blocking of Stat3 can repress procollagen gene expressions [19]. Some studies on the mechanism by which Stat3 exacerbates myocardial fibrosis showed that Stat3 is bound with COL1A1 and COL3A1 promoter and activates their transcription [30]. In this study, we demonstrated GF can contribute to increased phosphorylation of Stat3 into the nucleus. Moreover, the ability of the CaMKII inhibitor KN-93 can counteract the fibrotic remodeling induced by GF in type 2 diabetes, underscoring the functional importance of the CaMKII/Stat3 interaction in myocardial fibrosis. Of note, the relationship of CaMKII and Stat3 differs in different diseases. A study showed that KN-93 could down-regulated Stat3 aggravated myocardial microvessel remodelling [31]. Furthermore, cardiomyocyte-specific Stat3 deficiency was also shown to impair cardiac contractility in hypertensive mice [32]. Therefore, the CaMKII/Stat3 pathway may serve distinct roles in different diseases.

In our study, we found that glucose fluctuations in type 2 diabetes exacerbated myocardial fibrosis via the CaMKII/Stat3 pathway. However, there were also some limitations in our study. First, we only investigated the role of CaMKII/Stat3 in regulating myocardial fibrosis with the inhibitors at the cellular level instead of the animal level. Second, we only used the inhibitor KN-93 to explore the underlying mechanisms, and did not use other inhibitors of CaMKII or knockout animals.

## Conclusion

In summary, our present study reveals that CaMKII plays a pivotal role in myocardial fibrosis in type 2 diabetes with GF. Targeting the CaMKII-Stat3 pathway may protect the heart from myocardial fibrosis induced by GF in type 2 diabetes.

### Electronic supplementary material

Below is the link to the electronic supplementary material.

Supplementary Material 1

## Data Availability

The data used in this research can be made available by the corresponding author upon a reasonable request.

## Abbreviations

CaMKII: Ca^2+^/calmodulin‑dependent protein kinase II
Stat3: Signal transducer and activator of transcription 3
GF: Glucose fluctuations
SD: Sprague dawley
STZ: Streptozotocin
DM: Diabetes mellitus
T2DM: Type 2 diabetes mellitus
EF: Ejection fraction
FS: Fractional shortening
LVIDd: Left ventricular internal diameter at end-diastole
LVIDs: Left ventricular internal diameter at end-systole
HE: Hematoxylin and eosin
NRCFs: Neonatal rat cardiac fibroblasts
PBS: Phosphate buffered saline
FBS: Fetal bovine serum
HG: High glucose
SEM: Standard error of mean

## References

[1] Battelino T, Alexander CM, Amiel SA, Arreaza-Rubin G, Beck RW, Bergenstal RM (2023). Continuous glucose monitoring and metrics for clinical trials: an international consensus statement. Lancet Diabetes Endocrinol.

[2] Ceriello A, Prattichizzo F, Phillip M, Hirsch IB, Mathieu C, Battelino T (2022). Glycaemic management in Diabetes: old and new approaches. Lancet Diabetes Endocrinol.

[3] Wang Y, Zhou J, Qi W, Zhang N, Tse G, Li G (2023). Visit-to-visit variability in fasting blood glucose predicts the new-onset Heart Failure: results from two large Chinese cohorts. Curr Probl Cardiol.

[4] Zhang J, Yang J, Liu L, Li L, Cui J, Wu S (2021). Significant abnormal glycemic variability increased the risk for arrhythmias in elderly type 2 diabetic patients. BMC Endocr Disord.

[5] Li F, Zhang L, Shen Y, Liu HH, Zhang ZY, Hu G (2023). Higher glucose fluctuation is associated with a higher risk of Cardiovascular Disease: insights from pooled results among patients with Diabetes. J Diabetes.

[6] Zhang ZY, Dang SP, Li SS, Liu Y, Qi MM, Wang N (2022). Glucose fluctuations aggravate myocardial fibrosis via the nuclear factor-κB-mediated nucleotide-binding oligomerization domain-like receptor protein 3 inflammasome activation. Front Cardiovasc Med.

[7] Benchoula K, Mediani A, Hwa WE (2023). The functions of ca(2+)/calmodulin-dependent protein kinase II (CaMKII) in Diabetes progression. J Cell Commun Signal.

[8] Lebek S, Chemello F, Caravia XM, Tan W, Li H, Chen K (2023). Ablation of CaMKIIδ oxidation by CRISPR-Cas9 base editing as a therapy for cardiac Disease. Science.

[9] Reyes Gaido OE, Nkashama LJ, Schole KL, Wang Q, Umapathi P, Mesubi OO (2023). CaMKII as a therapeutic target in Cardiovascular Disease. Annu Rev Pharmacol Toxicol.

[10] Hegyi B, Bers DM, Bossuyt J (2019). CaMKII signaling in Heart Diseases: emerging role in diabetic cardiomyopathy. J Mol Cell Cardiol.

[11] Lu S, Liao Z, Lu X, Katschinski DM, Mercola M, Chen J (2020). Hyperglycemia acutely increases cytosolic reactive oxygen species via o-linked GlcNAcylation and CaMKII activation in mouse ventricular myocytes. Circ Res.

[12] Chen Y, Li X, Hua Y, Ding Y, Meng G, Zhang W. RIPK3-mediated necroptosis in diabetic cardiomyopathy requires CaMKII activation. Oxid Med Cell Longev 2021; 2021:6617816. 10.1155/2021/6617816 PMC8203407 34194608

[13] Das K, Basak M, Mahata T, Kumar M, Kumar D, Biswas S (2022). RGS11-CaMKII complex mediated redox control attenuates chemotherapy-induced cardiac fibrosis. Redox Biol.

[14] Wu X, Xu M, Geng M, Chen S, Little PJ, Xu S (2023). Targeting protein modifications in metabolic Diseases: molecular mechanisms and targeted therapies. Signal Transduct Target Ther.

[15] Chen Z, Zhou H, Huang X, Wang S, Ouyang X, Wang Y, et al. Pirfenidone attenuates cardiac hypertrophy against isoproterenol by inhibiting activation of the janus tyrosine kinase-2/signal transducer and activator of transcription 3 (JAK-2/STAT3) signaling pathway. Bioengineered. 2022;13(5):12772–82. 10.1080/21655979.2022.2073145 PMC9276057 35609321

[16] Huo S, Shi W, Ma H, Yan D, Luo P, Guo J et al. Alleviation of inflammation and oxidative stress in pressure overload-induced cardiac remodeling and heart failure via IL-6/STAT3 inhibition by raloxifene. Oxid Med Cell Longev 2021; 2021:6699054. 10.1155/2021/6699054 PMC8007383 33824698

[17] Zhuang L, Jia K, Chen C, Li Z, Zhao J, Hu J (2022). DYRK1B-STAT3 drives cardiac hypertrophy and Heart Failure by impairing mitochondrial bioenergetics. Circulation.

[18] Chen Q, Lv J, Yang W, Xu B, Wang Z, Yu Z (2019). Targeted inhibition of STAT3 as a potential treatment strategy for Atherosclerosis. Theranostics.

[19] Luo W, Huang L, Wang J, Zhuang F, Xu Z, Yin H (2019). Inhibition of EGFR-STAT3 attenuates cardiomyopathy in streptozotocin-induced type 1 Diabetes. J Endocrinol.

[20] Unudurthi SD, Nassal D, Greer-Short A, Patel N, Howard T, Xu X (2018). βIV-Spectrin regulates STAT3 targeting to tune cardiac response to pressure overload. J Clin Invest.

[21] Ying C, Zhou X, Chang Z, Ling H, Cheng X, Li W (2016). Blood glucose fluctuation accelerates renal injury involved to inhibit the AKT signaling pathway in diabetic rats. Endocrine.

[22] Wu W, Chai Q, Zhang Z (2021). Glucose fluctuation accelerates cardiac injury of diabetic mice via sodium-dependent glucose cotransporter 1 (SGLT1). Arch Biochem Biophys.

[23] Wang P, Xu S, Xu J, Xin Y, Lu Y, Zhang H (2022). Elevated MCU expression by CaMKIIδB limits pathological cardiac remodeling. Circulation.

[24] Zhang L, Li F, Liu HH, Zhang ZY, Yang F, Qian LL (2022). Glycaemic variability and risk of adverse cardiovascular events in acute coronary syndrome. Diab Vasc Dis Res.

[25] Ji M, Su L, Liu L, Zhuang M, Xiao J, Guan Y (2023). CaMKII regulates the proteins TPM1 and MYOM2 and promotes diacetylmorphine-induced abnormal cardiac rhythms. Sci Rep.

[26] Lebek S, Pichler K, Reuthner K, Trum M, Tafelmeier M, Mustroph J (2020). Enhanced CaMKII-dependent late I(na) induces atrial proarrhythmic activity in patients with sleep-disordered breathing. Circ Res.

[27] Wei X, Jin J, Wu J, He Y, Guo J, Yang Z, et al. Cardiac-specific BACH1 ablation attenuates pathological cardiac hypertrophy by inhibiting the Ang II type 1 receptor expression and the Ca^2+^/CaMKII pathway. Cardiovasc Res. 2023;119(9):1842–55. 10.1093/cvr/cvad086 37279500

[28] Zhang J, Liang R, Wang K, Zhang W, Zhang M, Jin L (2022). Novel CaMKII-δ inhibitor hesperadin exerts dual functions to ameliorate cardiac ischemia/reperfusion injury and inhibit Tumor growth. Circulation.

[29] Li Y, Liu X, Wan L, Han B, Ma S, Pan H (2023). Metformin suppresses cardiac fibroblast proliferation under high-glucose conditions via regulating the mitochondrial complex I protein Grim-19 involved in the Sirt1/Stat3 signaling pathway. Free Radic Biol Med.

[30] Qing Z, Yuan W, Wang J, Song W, Luo J, Wu X (2022). Verapamil inhibited the development of ureteral stricture by blocking CaMKII-mediated STAT3 and Smad3/JunD pathways. Int Urol Nephrol.

[31] Ni Y, Deng J, Bai H, Liu C, Liu X, Wang X (2022). CaMKII inhibitor KN-93 impaired angiogenesis and aggravated cardiac remodelling and Heart Failure via inhibiting NOX2/mtROS/p-VEGFR2 and STAT3 pathways. J Cell Mol Med.

[32] Altara R, Harmancey R, Didion SP, Booz GW, Zouein FA (2016). Cardiac STAT3 deficiency impairs contractility and metabolic homeostasis in Hypertension. Front Pharmacol.

