# Supplementary material for: Glucose fluctuations aggravate myocardial fibrosis via activating the CaMKII/Stat3 signaling in type 2 diabtetes
Source: Diabetol Metab Syndr. 2023 Oct 28;15:217. doi: 10.1186/s13098-023-01197-5 (PMC10612236; doi:10.1186/s13098-023-01197-5)

Original blots

Red boxes represent the location of target proteins;

Green boxes represent the representative blots used in the manuscript.

Figure 2B Collagen I + Collagen III + TGF-β1 + β-tubulin

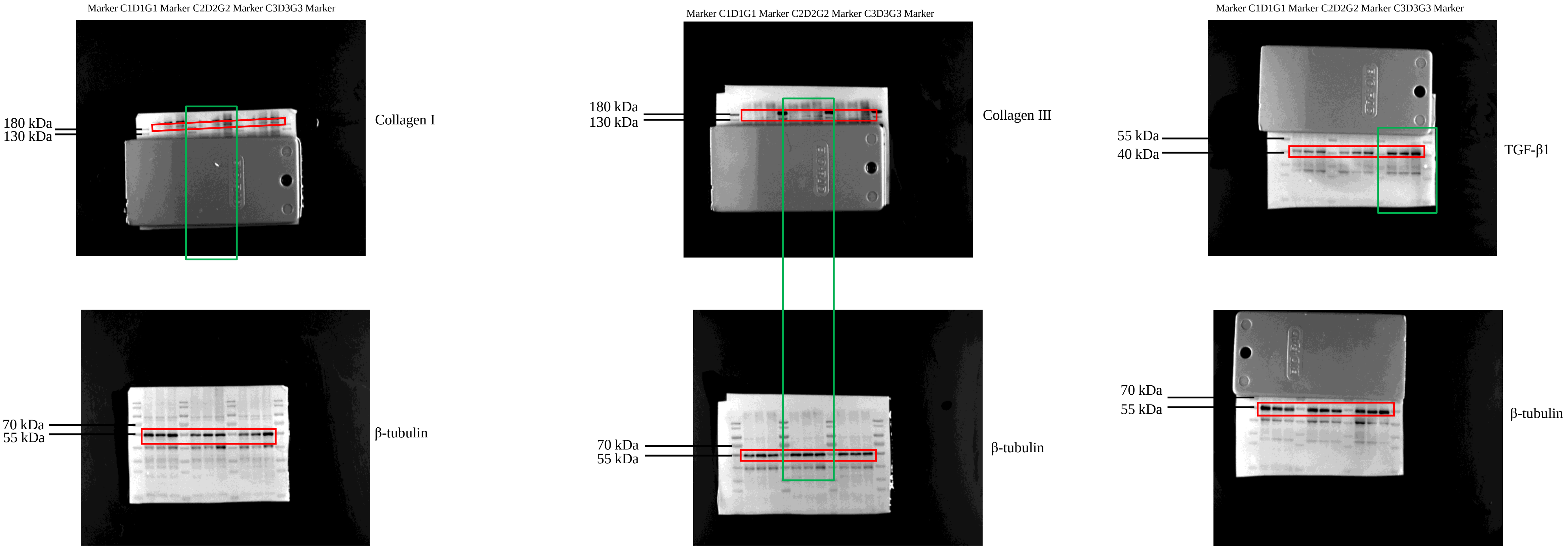

Figure 2G Collagen I + Collagen III + TGF-β1 + β-actin

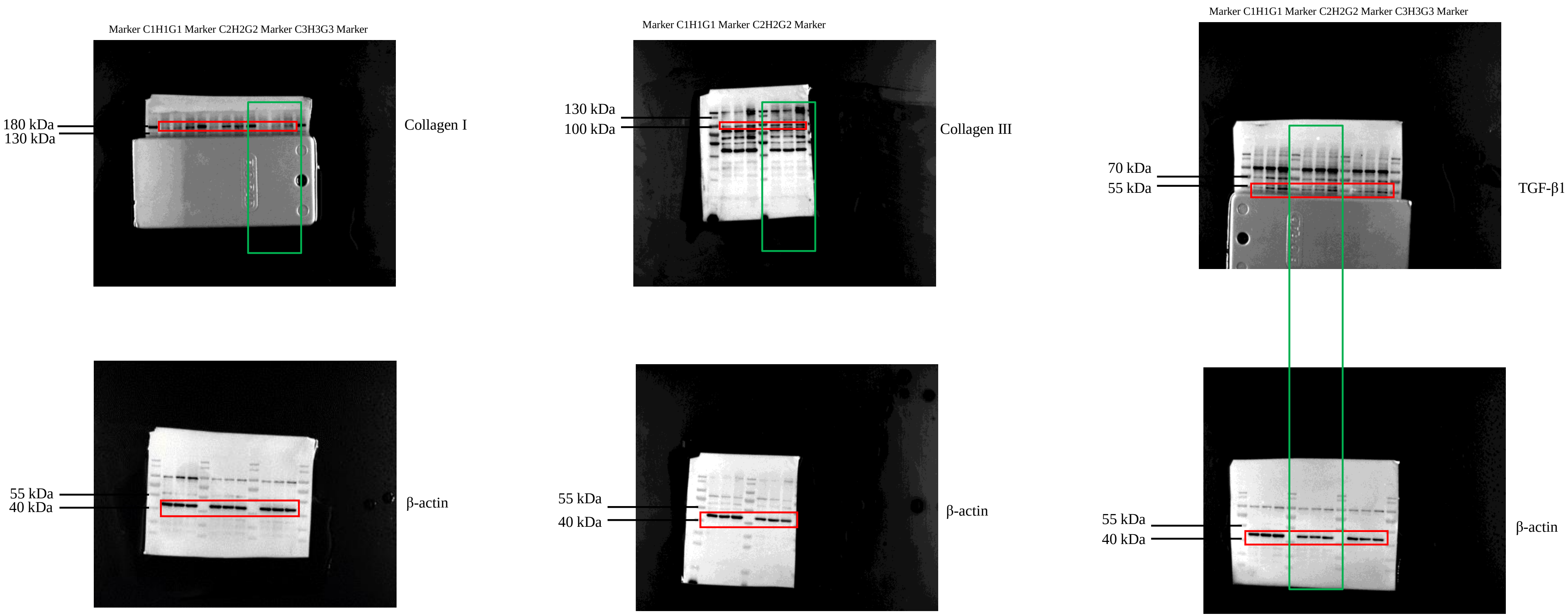

Figure 3A p-CaMKII+ CaMKII + p-Stat3+Stat3+ β-tubulin

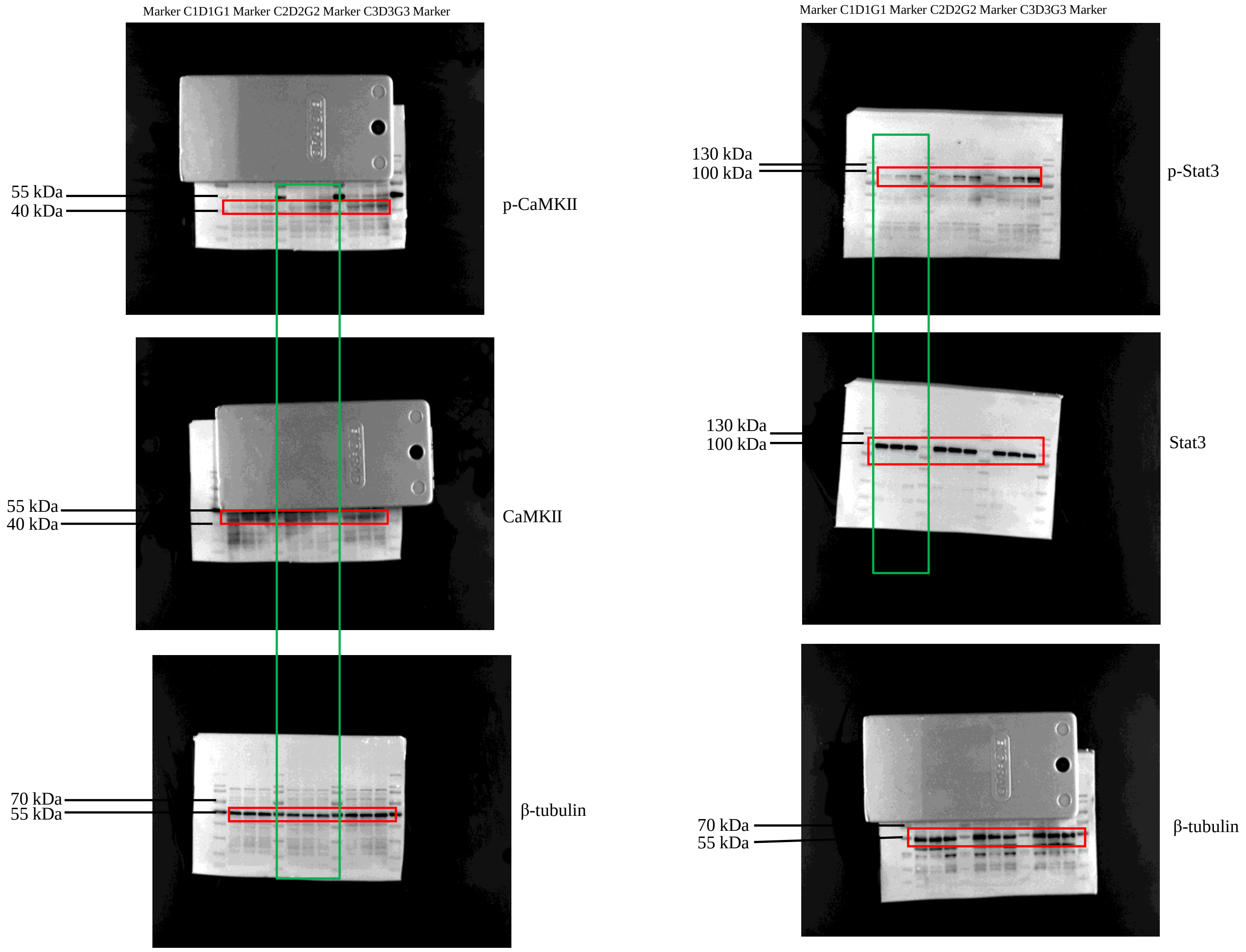

Figure 3D p-CaMKII+ CaMKII + p-Stat3+Stat3+ β-tubulin

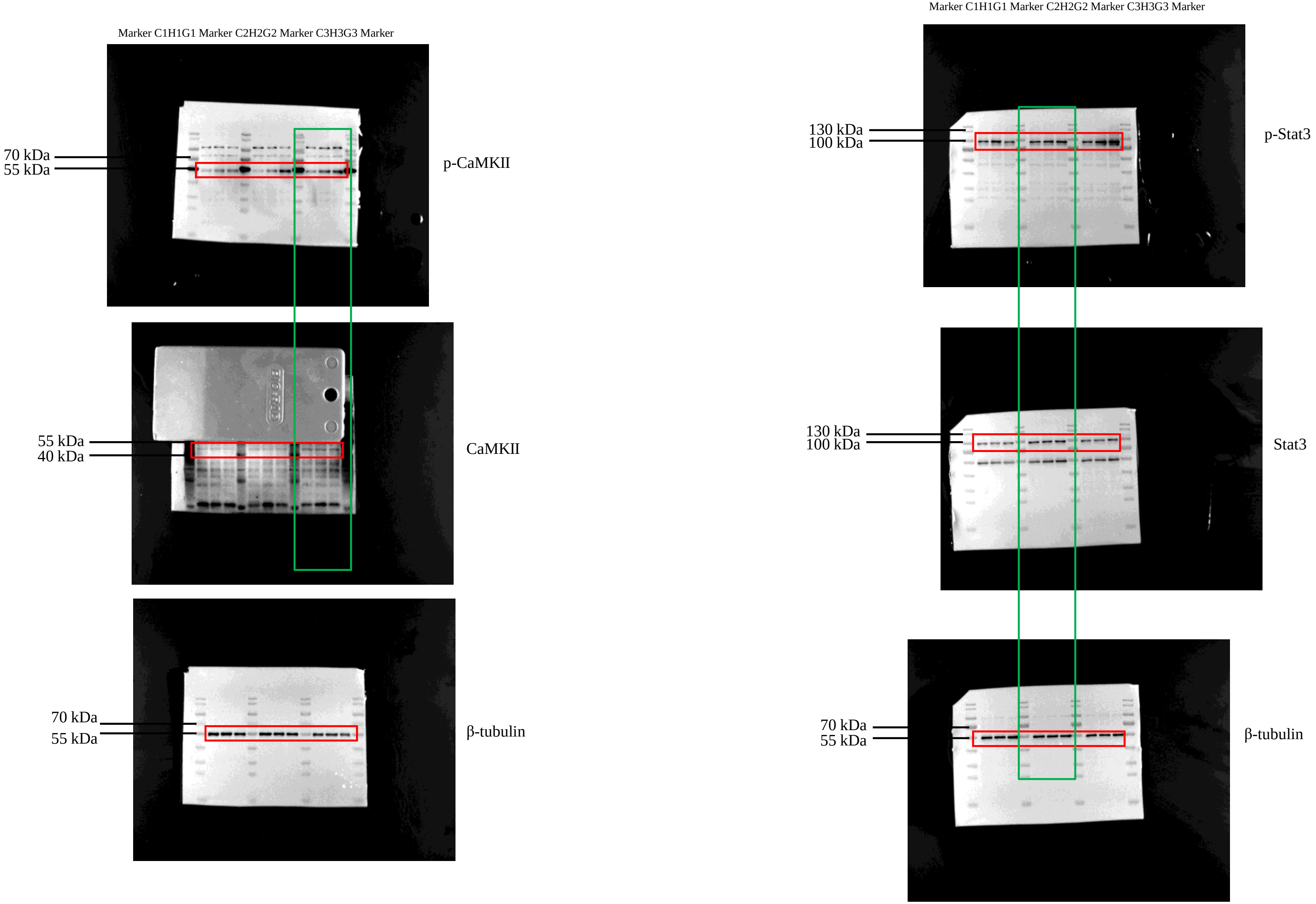

Figure 4A p-CaMKII+ CaMKII + p-Stat3+Stat3+ β-tubulin

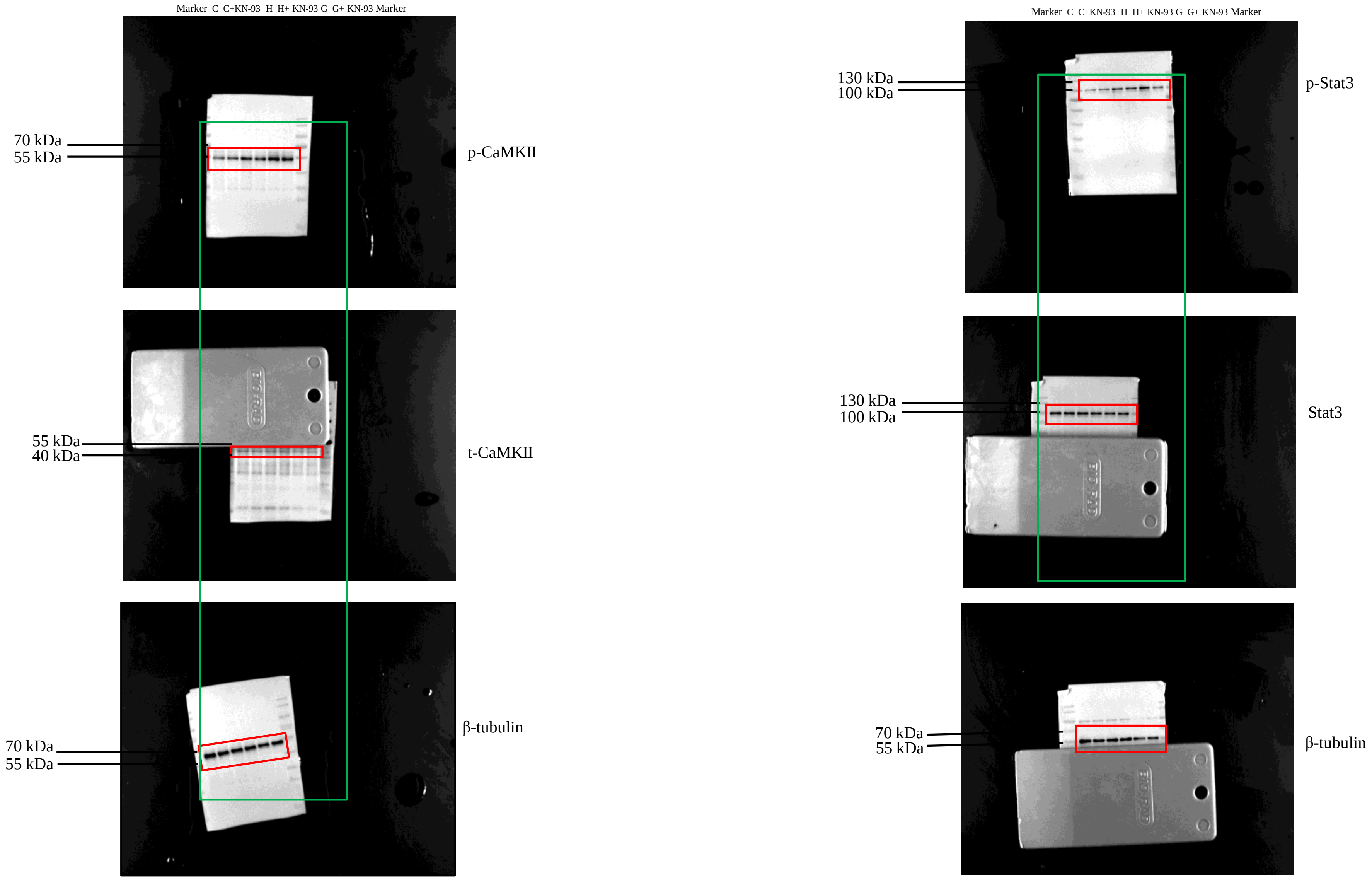

Figure 5A Collagen I + Collagen III + TGF-β1 + β-actin

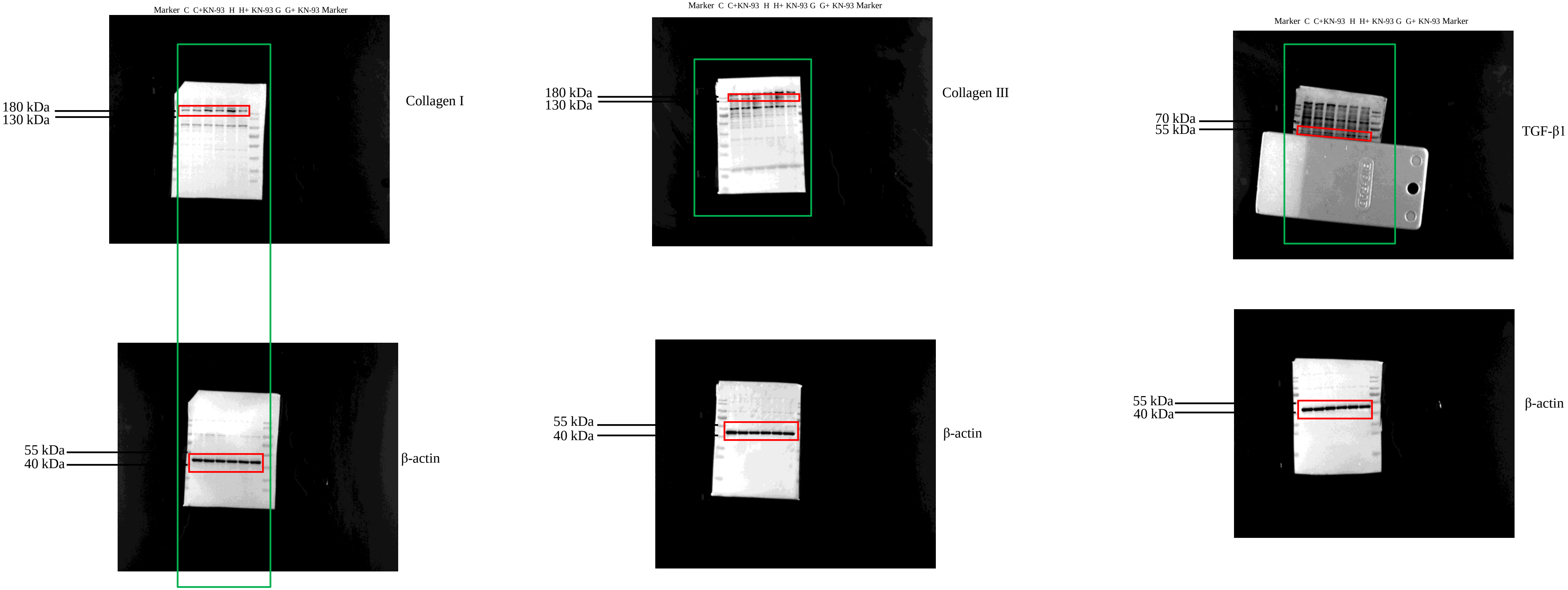

Supplement: Supplementary file 1 — Supplementary Material 1 [file 13098_2023_1197_MOESM1_ESM.pdf]
